# Editorial: Nutrients recycling in hydroponics: opportunities and challenges toward sustainable crop production under controlled environment agriculture, volume II

**DOI:** 10.3389/fpls.2025.1656162

**Published:** 2025-08-01

**Authors:** Md Asaduzzaman, Toshiki Asao

**Affiliations:** ^1^ Department of Environmental and Bioresource Sciences, Faculty of Bioenvironmental Sciences, Kyoto University of Advanced Science, Kyoto, Japan; ^2^ Laboratory of Horticultural Science, Department of Agricultural Science and Technology, Faculty of Agriculture, Setsunan University, Osaka, Japan

**Keywords:** hydroponic nutrient recycling, LED and HPS supplementation, zinc biofortification, nutrient interaction, physiological and biochemical responses, reuse of soilless substrate, controlled environment agriculture (CEA)

## Hydroponic nutrient recycling

In controlled environment agriculture (CEA), hydroponic nutrient solution is essentially recycled to increase resource use efficiency and environmental sustainability (Rufí-Salís et al., 2020). This recycling approach also opens up opportunities to manage other inputs that affect the overall outcome of the crop. In this context, adjusting the nutrient composition, fine-tuning the growing environment, and applying the latest CEA technologies can make a big difference. These strategies can improve the quality of the produce, enhance levels of antioxidants and other bioactive compounds, and support the biofortification of essential micronutrients (Asaduzzaman et al., 2018; Son et al., 2020; Ciriello et al., 2021).

In recycled hydroponics, nutrient solutions are collected and reused in successive cultures which is common in CEA, like plant factories with LED lighting. However, the accumulation of inhibitory root exudates in the solution can cause allelochemical stress and negatively impact the growth of crops such as lettuce, strawberries, and cucumbers (Asao et al., 2004; Lee et al., 2006; Kitazawa et al., 2005; Yu and Matsui, 1994). A leading research group has conducted extensive studies on mitigating autotoxicity in recycled hydroponic systems using various approaches (Lee et al., 2006; Kitazawa et al., 2005; Mondal et al., 2013; Kitazawa et al., 2007; Asaduzzaman et al., 2012; Talukder et al., 2019). Despite these efforts, there is still a need for more precise and effective methods to fully resolve this issue.

This Research Topic brings together five original research articles that highlights recent advances in the use of artificial light (AFL) in plant science. It also focuses on key areas including nutrient bioaccumulation, zinc biofortification, nutrient concentration, and how light quality affects physiological and biochemical responses in plants. Additionally, one article discusses the reuse of soilless substrates as a sustainable alternative to synthetic substrate in commercial strawberry production.

## Supplemental LED and HPS influence basil yield, nutrients, and resource efficiency

Light quantity, quality, and duration affect plant growth and development under CEA. Using LEDs, growers can optimize yield and quality by adjusting these factors based on real-time monitoring. Aligning supplemental lighting with seasonal sunlight may boost light use efficiency and reduce energy costs.

Hammock et al. investigated the supplementation of LED and high-pressure sodium (HPS) lights at different daily light integrals (DLI) to enhance winter greenhouse basil production. Light use efficiency (LUE) were measured along with fresh biomass, and nutrient uptake. Nine lighting conditions were used viz. one non-supplemented natural light (NL) as control, two end-of-day (EOD) HPS light applied for 6 and 12 hours; five EOD LED lights with red: blue (R20:B80) spectrum applied for 3, 6, 9, 12, and 18 hours, and one continuous LED light for 24 hours. The intensity of each supplemental lighting (SL) was 100 μmol·m^-2^·s^-1^ with average DLI of 9.9 mol·m^-2^·d^-1^ throughout the entire growth period. It was reported that both supplemental lighting and growing season had significant impacts on biomass production and nutrient accumulation. However, lower yield was recorded in some SL than that of the non-supplemented NL plants. Among the different seasons, January produced the lowest fresh and dry mass of basil, while November yielded the highest. Mineral analysis showed that both lighting type and season influenced macro- and micronutrient uptake in plant parts. Additionally, LUE varied widely among light conditions applied. In this study, plant quality or secondary metabolites were not evaluated, which are important for consumer view point. Future research was suggested to address the above research work using metabolomic and transcriptomic tools that can explore how different lighting strategies impact plant physiology and biochemical pathways.

## Zinc biofortification of hydroponically grown basil

Herbs, medicinal and aromatic plants are the riches source of antioxidants and bioactive compounds provides human health benefits. Biofortification of micronutrients including Zn in hydroponically grown crops including basil provides a practical and sustainable means.

Ciriello et al. applied four concentration of Zn such as 12.5, 25.0, 37.5, and 50 μM in the hydroponic nutrient solution to evaluate yield and quality, physiological attributes, and Zn bioaccumulation in basil. Two basil cultivars namely ‘Aroma 2’ and ‘Eleonora’ were cultivated in floating raft hydroponic system. It was reported that yield was significantly reduced with the increased concentration of Zn in hydroponic nutrient solution but this trend was less evident in case of variety ‘Aroma 2’. Plants grown in 50 μM Zn produced increased concentration of carotenoids, polyphenols, and antioxidant activity by 19.76%, 14.57%, and 33.72%, respectively, compared to the control. Strong positive correlation between Zn concentrations in the hydroponic nutrient solution and its bioaccumulation in basil plant recommend as an effective approach of Zn biofortification. In this study, cultivar-dependent response results highlight the importance of variety selection in biofortifying non-hyperaccumulating crops to enrich micronutrients without reducing yield. It also mentioned that biofortified Genovese basil can enhance Zn intake while also delivering beneficial phytochemicals like carotenoids and polyphenols.

## Nitrogen and light quality modulate plant growth and resource allocation

CEA enables year-round and high-yield production is the key for high value horticultural crops grown hydroponically. Light quality and quality with nitrogen supply are the vital consideration in successful crop cultivation system. Significant research efforts have given on the sole influence nitrogen and light quality on plant growth, dry matter accumulations and nitrogen uptake in plants growth in hydroponics.

Liang et al. studied three light conditions viz. 100% red light, R; 50% red light + 50% blue light, RB; 100% blue light, B and either low or high nitrogen concentrations as 0.1 mM N, LN and 10 mM N, HN on the growth, dry matter accumulation, and nitrogen content in lettuce. Shoot dry weight was found to be increased in red light compared to both B and RB, irrespective of either LN or HN, while 100% blue light, B enhance root growth even under LN condition. It was evident that lower allocation of nitrogen to leaf compared to B under LN, but similar leaf allocation under HN. Shoot nitrate nitrogen content found to be increased in both nitrogen levels under B up to 14 hours with HN, while slightly differed with light quality under LN. It also reported that nitrogen content and nitrogen nutrition index, NR activity was increased in plants exposed to B. Therefore, interaction between B and nitrogen application control the dry matter and nitrogen allocation between shoot and root. Results of this study showed promise in the application and design of nitrogen supply and light environments for CEA.

## Physiological and biochemical responses of halophyte to NaCl concentrations

*Limonium tetragonum* (Thunb.) A. A. Bullock, a halophytic medicinal plant native to Korea’s southwest coast, exhibits various pharmacological effects. Its salt defense mechanisms believed to stimulate the production of secondary metabolites and functional compounds.

Jang et al. investigated optimal NaCl levels for enhancing these metabolites in *L. tetragonum* grown in hydroponics. Seedlings cultivated for 3 weeks were treated with 0, 25, 50, 75, and 100 mM NaCl in Hoagland’s solution for 8 weeks. Results revealed that growth and chlorophyll fluorescence were unaffected at NaCl levels below 100 mM, though leaf water potential declined with increasing salinity. Na^+^ accumulation in aerial tissues increased sharply, while antagonistic K^+^ levels decreased. Total amino acid content decreased compared to 0 mM, but levels of urea, proline, β-alanine, ornithine, and arginine increased with higher salinity. At 100 mM NaCl, proline comprised 60% of total amino acids, suggesting its key role in osmoregulation. Flavonoids were the dominant compounds identified, with a flavanone detected only under NaCl treatments. Four myricetin glycosides significantly increased compared to 0 mM, indicating that salinity enhances their biosynthesis. The study concluded that 75 mM NaCl is optimal for boosting secondary metabolite production in *L. tetragonum*.

## Reuse of plant based soilless substrate for strawberry production

Greenhouse and polytunnel strawberry production are gaining popularity worldwide.

Woznicki et al. investigated the reuse of two commonly used soilless substrates such as coir and peat, as well as stand-alone wood fiber from Norway spruce, a promising alternative substrate. Their study evaluated the feasibility of reusing these substrates over multiple production cycles. Peat, coir, and wood fiber all supported successful strawberry growth, even after two cycles. While peat and wood fiber produced the highest yields in the first year, yields declined slightly in subsequent uses, though they remained comparable to both new and reused coir. In particular, strawberries grown in wood fiber accumulated more sugars and produced taller plants. Wood fiber also showed the highest nitrogen accumulation over three production cycles, while all substrates showed a decline in potassium. Although wood fiber initially had the highest cellulose content, it decreased over time to levels similar to coir and peat. The findings suggest that both wood fiber and substrate reuse are viable strategies for more sustainable strawberry production in soilless systems.
